# Spontaneous endometriosis in rhesus macaques: evidence for a genetic association with specific *Mamu-A1* alleles

**DOI:** 10.5194/pb-4-117-2017

**Published:** 2017-06-22

**Authors:** Ivanela Kondova, Gerco Braskamp, Peter J. Heidt, Wim Collignon, Tom Haaksma, Nanine de Groot, Nel Otting, Gaby Doxiadis, Susan V. Westmoreland, Eric J. Vallender, Ronald E. Bontrop

**Affiliations:** 1 Animal Science Department, Division of Pathology and Microbiology, Division of Veterinary care, Biomedical Primate Research Centre, 2288 GJ Rijswijk, the Netherlands; 2 Department of Comparative Genetics, Biomedical Primate Research Centre, 2288 GJ Rijswijk, the Netherlands; 3 AbbVie Bioresearch Center, Immunology, Pharmacology, Pathology and Exploratory Toxicology, Worcester, MA 01605, USA; 4 Department of Psychiatry and Human Behavior, University of Mississippi Medical Center, Jackson, MS 39216, USA; 5 Division of Veterinary Medicine, Tulane National Primate Research Center, Covington, LA 70433, USA; † deceased

## Abstract

Endometriosis is a poorly understood common debilitating women's reproductive disorder resulting from proliferative and ectopic endometrial tissue associated with variable clinical symptoms including dysmenorrhea (painful menstrual periods), dyspareunia (pain on intercourse), female infertility, and an increased risk of malignant transformation. The rhesus macaque (*Macaca mulatta*) develops a spontaneous endometriosis that is very similar to that seen in women. We hypothesized that specific major histocompatibility complex (MHC) alleles may contribute to the pathogenesis of endometriosis. As part of a collaboration between the Biomedical Primate Research Centre (BPRC) in the Netherlands and the New England Primate Research Center (NEPRC) in the United States, we analyzed DNA sequences of MHC class I (*Macaca mulatta, Mamu-A1*) and class II (*Mamu-DRB*) alleles from rhesus macaques with endometriosis and compared the allele frequencies with those of age-matched healthy macaques. We demonstrate that two MHC class I alleles are overrepresented in diseased macaques compared to controls: Mamu-A1*001, 33.3 % in BPRC animals with endometriosis vs. 11.6 % in healthy macaques (p= 0.007), and Mamu-A1*007, 21.9 % NEPRC rhesus macaques vs. 6.7 %, (p= 0.003). We provide evidence that select MHC class I alleles are associated with endometriosis in rhesus macaques and suggest that the disease pathogenesis contribution of MHC class I warrants further research.

## 1 Introduction

### 1.1 Endometriosis in humans

Endometriosis is a chronic debilitating inflammatory disease that affects approximately 10 to 20 % of women of reproductive age and roughly 50 % of women with infertility (Giudice, 2010). The associated clinical symptoms like dysmenorrhoea, dyspareunia, and chronic pelvic pain have a negative impact on the quality of life of women affected with the disorder (Gupta et al., 2008). Histologically, the disease is defined by the presence of endometrial glandular and stromal tissue in organs and tissues outside of the uterine endometrium.

Experimental studies in women are hindered by the risks and complications associated with repetitive biopsy or surgical procedures. As a consequence, the pathogenesis of endometriosis remains incompletely understood. It is likely that endometriosis is a complex and multifactorial disorder triggered by hormonal, immunologic, genetic, and environmental factors. One hypothesized process in the pathogenesis of endometriosis is metaplasia, involving the transformation of tissues in the peritoneal cavity into endometrial tissue driven by hormonal or immunological factors (Sourial et al., 2014). Hormones play multiple roles with estrogen promoting proliferation of endometrial lesions and progesterone limiting endometrial proliferation. Inflammation, immune dysregulation, and oxidative stress have also been associated with endometriosis, contributing to cytokine-mediated endometrial growth (Forte et al., 2014). Other hypothesized processes include the suppression of normal apoptosis of endometrial glandular cells, proliferation of a population of progenitor or stem cells, epigenetic alterations (Forte et al., 2014), and the oldest theory of retrograde menstruation (Sampson, 1927).

Hereditary studies in women with endometriosis showed increased incidence in relatives of affected women compared to women without a familial history of endometriosis (Simpson et al., 1980). Consequently, the role of genetics in endometriosis has been long hypothesized (Moen et al., 1984; Simpson et al., 1984; Kennedy, 1999; Ishii et al., 2003; Bischoff and Simpson, 2004; Zondervan et al., 2001, 2004), but, like many complex diseases, specific causative genes or haplotypes have been elusive. In addition, while early studies implicated an immunologic basis for endometriosis (Steele et al., 1984), there was no identified association with the human leukocyte antigen (HLA), which are the genes encoding the major histocompatibility complex (MHC) in humans (Moen et al., 1984; Simpson et al., 1984). More recent studies have been increasingly suggestive of the role of immune dysfunction and inflammation in endometriosis (Ahn et al., 2016; Yamada-Nomoto et al., 2016), but, while there have been more suggestions of an association with HLA in endometriosis (Ishii et al., 2003; Kitawaki et al., 2002), possibly in concert with specific killer immunoglobulin-like receptor (KIR) genotypes (Kitawaki et al., 2007; Nowak et al., 2015), the role of the HLA/MHC remains an open question.

### 1.2 Rhesus macaque as a model for endometriosis

Controlled experiments in humans are difficult due to limitations on repeated imaging and surgical biopsies for disease monitoring (Story and Kennedy, 2004). Therefore, animal models provide an invaluable tool for studying complex diseases like endometriosis. Although the use of rodent models of endometriosis has some advantages with respect to genetic manipulation and affordability, these species differ greatly from humans, making comparisons difficult. Baboons and macaques have been the best nonhuman primate (NHP) models to study endometriosis' pathogenesis, pathophysiology, spontaneous evolution, and new medical treatment options (D'Hooghe et al., 2009; Fazleabas et al., 2002; Yamanaka et al., 2012). In fact spontaneous endometriosis only occurs in humans and menstruating NHPs. Rhesus macaques share many similarities with humans, such as their reproductive physiology, which is of particular relevance. Menarche in rhesus monkeys occurs at about 3 years of age, the length of the menstrual cycle is about 28 days, and menstrual bleeding lasts for about 4 days (Catchpole and van Wagenen, 1975). As in women, studies have implicated genetic predisposition to endometriosis in macaques (Zondervan et al., 2001, 2004). Humans and rhesus macaques have a comparable major histocompatibility complex (also known as the human leukocyte antigen in humans) with two main antigen-presenting classes of molecules. In rhesus macaques MHC *(MhcMamu)* class I consists of *Mamu-A* and *Mamu-B* and class II of *Mamu-DR*, *Mamu-DQ*, and *Mamu-DP* molecules. In both species, the genes encoding both MHC I and II molecules are characterized by high allelic variation, but, while macaques show a high degree of copy number variation of class I and II, specifically *Mamu-B* genes, the equivalent of the human C gene is absent. The aim of our study is to examine any genetic susceptibility of MHC alleles to endometriosis in two colonies of rhesus macaques.

## 2 Materials and methods

### 2.1 Description of the colonies

The Biomedical Primate Research Centre (BPRC) in Rijswijk, the Netherlands, is fully accredited by the Association for Assessment and Accreditation of Laboratory Animal Care (AAALAC) and maintains a breeding colony of approximately 1100 rhesus macaques (*Macaca mulatta*). Animals are conventionally housed in large social breeding groups (one alpha male with several adult females and their juvenile and adolescent offspring), mimicking the natural ecology. The housing of these groups consists of interconnected indoor (72 m${}^{2})$ and outdoor (208 m${}^{2})$ enclosures with elevated sitting locations and enrichment devices (Vernes and Louwerse, 2010). Animals are fed on a diet of commercially available monkey chow, fruits, vegetables, and grains. Water is available ad libitum. Housing and care is in accordance with the Dutch law on animal experimentation, which follows EU Directive 86/609/EEC. The coefficient of inbreeding is calculated annually for all breeding animals according to Wrigh (1922), and the parentage is defined for all newborns by means of STR typing with 24 microsatellites localized on 16 different chromosomes.

**Figure 1:**
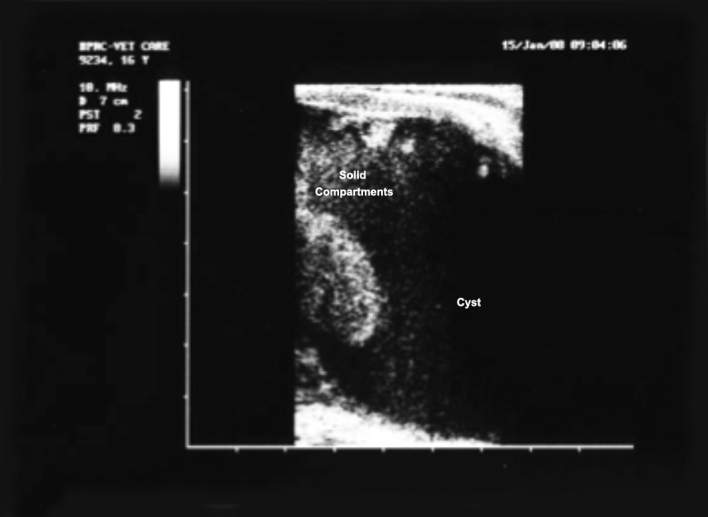
Ultrasound image of endometrial lesions of a 16-year-old rhesus macaque no. 9234 from the BPRC breeding colony. Image was taken with a flat probe, and it shows a cystic lesion with solid compartments.

At the time of data collection, the New England Primate Research Center (NEPRC) was a specific-pathogen-free colony of approximately 2000 primates maintained in accordance with federal and institutional guidelines mandated by the Institutional Animal Care and Use Committee (IACUC) of Harvard Medical School and accredited by the AAALAC. Rhesus macaques were housed in harems including one male and several adult females with pre-weaning offspring. Colony rooms were on a 12 h light–dark cycle, and the animals received a diet of monkey chow (Harlan Teklad monkey diet) supplemented with fresh fruit. A variety of enrichment objects were available at all times. All animal procedures including euthanasia were performed in accordance with guidelines and recommendations of the Committee on Animals of Harvard Medical School and the National Institutes of Health Guide for the Care and Use of Laboratory Animals (publication no. 85-23, revised 1996). Research protocols were approved by the Harvard Medical School Animal Care and Use Committee.

Both BPRC and NEPRC maintained complete medical records and familial relationships on all colony animals. After death, all animals were necropsied within several hours of death, often immediately following euthanasia, and representative sections of tissues were collected, flash frozen, and stored at -80 ${}^{\circ }$C, as well as fixed in 10 % neutral buffered formalin (NBF) and embedded in paraffin. The records from gross and histopathological examinations were held on the computerized database.

**Figure 2:**
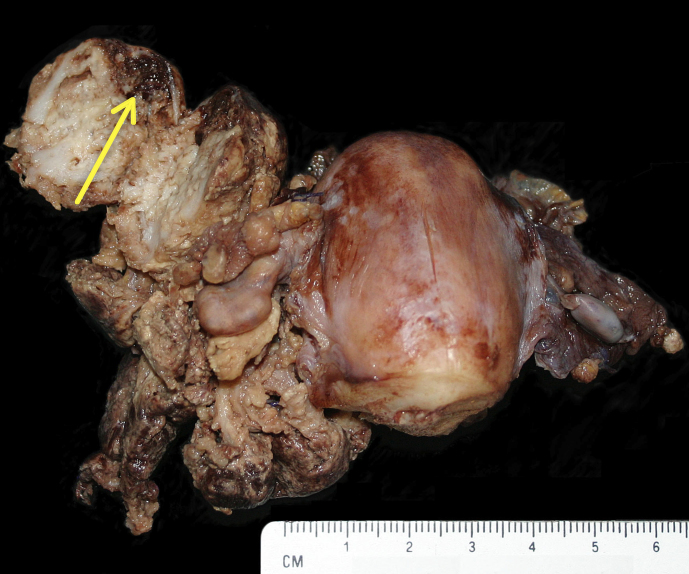
Gross pathology of uterus from rhesus macaque with endometriosis. The ovaries and fallopian tubes are embedded and distorted by accumulation of solid fibrous masses (scar tissue) and formation of endometrial cysts (arrow shows an open large cyst with yellow fibrous nodules in the center and dark red-brown fluid seen at the edge).

### 2.2 Identification of animals with endometriosis and case selection for the study

**Figure 3:**
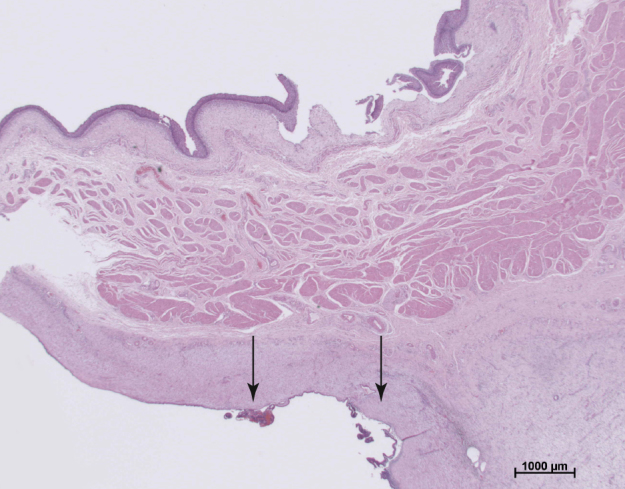
Urinary bladder of rhesus macaque with endometrial lesions. The serosa is infiltrated by endometrial glands, endometrial stroma, and inflammatory cells (hematoxylin and eosin staining).

Information on the presence of endometriosis was obtained through necropsy reports and archived gross, histological, and diagnostic representative images shown in Figs. 1, 2, and 3. The main criteria for the animals selected for the study were the clinically (bloating, pain, dysmenorrhea, ultrasound-detected cystic lesions) and histologically proven endometriosis (ectopic proliferative endometrial glandular and stromal tissues, hemosiderin, and hemorrhage). Necropsy records from NEPRC were examined to identify female rhesus macaques (*Macaca mulatta*) over 1 year of age for which representative tissues from all organs had been collected and examined histologically by routine hematoxylin and eosin staining. Cases with a diagnosis of endometriosis were reviewed and selected if frozen endometrial tissues were archived. A similar selection of tissues was made from the tissue bank at BPRC (Table 1). Control or unaffected animals were defined as such according their full necropsy report providing evidence of absence of endometriosis. All animals with endometriosis were of Indian origin, except animals 8612 and BB93, which are Indian × Burmese and Indian × Chinese mixed-breed animals, respectively, and animal 4050, which is of Burmese origin. The control animals from the two colonies were of Indian origin.

**Table 1:** Cohorts of rhesus macaques with endometriosis housed at NEPRC and BRPC shown with age, body weight, and surgical history of caesarian sections.

	Animal	Necrop. no.	Species	Sex	Source	Age (years)	Weight (kg)	Sample	C-section
1	148-90	A06-313	*M. mulatta*	F	NEPRC	16	11.1	DNA	no C-section
2	183-90	A06-314	*M. mulatta*	F	NEPRC	16	10.7	DNA	no C-section
3	265-87	A06-316	*M. mulatta*	F	NEPRC	19	11.6	DNA	no C-section
4	196-89	A06-317	*M. mulatta*	F	NEPRC	17	10.2	DNA	no C-section
5	256-89	A06-318	*M. mulatta*	F	NEPRC	17	9.4	DNA	no C-section
6	236-88	A06-323	*M. mulatta*	F	NEPRC	18	8.3	DNA	no C-section
7	536-91	A06-324	*M. mulatta*	F	NEPRC	15	10.2	DNA	no C-section
8	169-92	A06-325	*M. mulatta*	F	NEPRC	14	11	DNA	no C-section
9	369-92	A06-326	*M. mulatta*	F	NEPRC	14	6.2	DNA	no C-section
10	103-87	A06-327	*M. mulatta*	F	NEPRC	19	7.9	DNA	C-section
11	259-87	A06-342	*M. mulatta*	F	NEPRC	19	8.3	DNA	no C-section
12	419-91	A06-352	*M. mulatta*	F	NEPRC	15	9.4	DNA	C-section
13	170-87	A07-2	*M. mulatta*	F	NEPRC	19	9	DNA	no C-section
14	142-92	A07-9	*M. mulatta*	F	NEPRC	14	10.8	DNA	C-section
15	229-87	A07-10	*M. mulatta*	F	NEPRC	19	8.6	DNA	no C-section
16	127-86	A07-36	*M. mulatta*	F	NEPRC	20	10.8	DNA	no C-section
17	468-87	A07-37	*M. mulatta*	F	NEPRC	19	15.3	DNA	2 C-sections
1	8803	06-1120	*M. mulatta*	F	BPRC	18	6	DNA	no C-section
2	1WQ	06-1129	*M. mulatta*	F	BPRC	21	7.8	DNA	no C-section
3	8851	06-1183	*M. mulatta*	F	BPRC	17	7.3	DNA	no C-section
4	8612	05-1047	*M. mulatta*	F	BPRC	18	8.41	DNA	no C-section
5	9250	05-1070	*M. mulatta*	F	BPRC	12	7.66	DNA	no C-section
6	8930	05-1107	*M. mulatta*	F	BPRC	16	6.97	DNA	no C-section
7	BB93	07-1386	*M. mulatta*	F	BPRC	16	10.35	DNA	no C-section
8	4050	07-1354	*M. mulatta*	F	BPRC	18	5.6	DNA	no C-section

### 2.3 Pedigree analysis of macaques from BRPC and NEPRC

Parental relationships between animals were determined from veterinary records. For most matings only a single sire was present at the time of conception. MHC transmission between parent and offspring was used to confirm relationships with further genetic tests when warranted. There were no ambiguous parentage calls among the animals involved in these studies.

**Table 2:** **(a)** *Mamu-A1* and *Mamu-DRB* genotypes of animals diagnosed with endometriosis and their family relationship. A question mark for *Mamu-A1* typing indicates that the animal is most probably homozygous for *Mamu-A1*. A question mark in the column “remarks” indicates that sharing of a MHC haplotype is possible but cannot be confirmed. Alleles in bold represent those which are present at a higher frequency in rhesus macaques with endometriosis than in healthy animals. **(b)** Allele frequencies for animals with endometriosis as well as colony frequencies for NEPRC and BPRC are shown. Significant p<0.05 values are bold.

**(a)**	Animal	Mamu-A1*	*Mamu-DRB*	Source	Remarks
1	148-90	*008/****007***	*W3:03/*W3:03	NEPRC	
2	183-90	*012/002*	1*03:03/3*04:10	NEPRC	
3	265-87	*004/002*	1*03:10/1*03:09	NEPRC	same father as 229 and 536; shared MHC?
4	196-89	***007/001***	1*04:06/*W6:06	NEPRC	same father as 256; no MHC sharing
5	256-89	*008/002*	1*03:03/1*03:09	NEPRC	same father as 196; no MHC sharing
6	236-88	*008****/007***	1*03:03/1*03:18	NEPRC	shared 1st MHC hapl. with 142; 2nd with 259
7	536-91	*012/008*	*W3:03/1*03:09	NEPRC	same father as 229 and 265; shared MHC?
8	169-92	*026/026*	1*04:06/1*03:09	NEPRC	shared MHC with 127
9	369-92	***007***/?	1*04:06/1*07:01	NEPRC	
10	103-87	*008/****007***	1*03:03/1*03:09	NEPRC	
11	259-87	***007/007***	*W6:06/1*03:18	NEPRC	shared 2nd MHC haplotype with 236
12	419-91	*004/008*	1*03:10/1*03:06	NEPRC	
13	170-87	*004/002*	1*03:09/*W3:03	NEPRC	
14	142-92	*008****/001***	1*03:03/*W3:03	NEPRC	shared 1st MHC hapl. with 236
15	229-87	*004/****001***	1*04:06/*W3:03	NEPRC	same father as 265 and 536; shared MHC?
16	127-88	*004/026*	1*03:09/1*03:17	NEPRC	shared MHC with offspring 169
17	468-87	*003/?*	1*04:04/1*03:06	NEPRC	
1	8803	*011/008*	3*04:10/1*04:06	BPRC	
2	1WQ	***001****/008*	1*04:06/1*03:03	BPRC	
3	8851	***001****/002*	*W6:06/1*04:06	BPRC	sibling of 8612; 1 shared MHC haplotype
4	8612	***001/007***	*W6:06/4*01:02	BPRC	sibling of 8651; 1 shared MHC haplotype
5	9250	***001****/004*	1*03:09/1*03:09	BPRC	sibling of 8930; 1 shared MHC haplotype
6	8930	***001/****002*	1*03:09/1*04:03	BPRC	sibling of 9250; 1 shared MHC haplotype
7	BB93	*008/?*	1*03:03/1*04:06	BPRC	
8	4050	*008/050*	1*03:21/*W26:04	BPRC	

**(b)**	NEPRC				BPRC			
*Mamu-A1*	Endom. (n)	Colony (n)	p val	$P_{\text{adj}}$	Endom. (n)	Colony (n)	p val	$P_{\text{adj}}$
A1*001	9.4 % (3)	11.9 % (90)	1	1	**33.3 % (5)**	**11.6 % (321)**	**0.007**	**0.04**
A1*002	12.5 % (4)	12.0 % (91)	1	1	13.3 %(2)	15.8 % (437)	1	1
A1*003	3.1 % (1)	0.3 % (2)	0.117	0.93	0 %(0)	4 % (111)	1	1
A1*004	15.6 % (5)	17.6 % (134)	1	1	6.7 %(1)	23.2 % (642)	0.251	1
A1*007	**21.9 % (7)**	**6.7 %(51)**	**0.003**	**0.03**	6.7 % (1)	4.7 % (130)	0.492	1
A1*008	21.9 % (7)	23.1 % (176)	1	1	26.7 %(4)	20.4 % (564)	0.475	1
A1*011	0 % (0)	0.2 % (1)	1	1	6.7 % (1)	2.7 % (75)	0.322	1
A1*012	6.3 % (2)	6.8 % (52)	1	1	0 % (0)	5.6 % (155)	1	1
A1*026	9.4 % (3)	1.5 % (11)	0.013	0.14	0 %(0)	1.1 % (30)	1	1
Total	100.0 % (32)	100.0 % (760)			100.0 % (15)	100.0 % (2766)		

$P_{\text{adj}}$ (p-adjusted) is the significance value after Bonferroni correction for multiple tests (see methods); “Endom.” represents animals with endometriosis; n is the number of alleles.

### 2.4 DNA extraction

Uterine tissue from NEPRC study animals was frozen in liquid nitrogen and pulverized. The powdered tissue was resuspended in digestion buffer and digested with proteinase K at 55 ${}^{\circ }$C overnight. DNA was isolated via phenol/chloroform extraction followed by ethanol precipitation. DNA pellets were resuspended in TE buffer, and sample concentration was measured via UV spectrometry at 260 nm. DNA isolation of BPRC's animals was performed on fresh EDTA blood or frozen peripheral blood mononuclear cells (PBMCs) by a standard salting-out method (Doxiadis et al., 2013) or by using the QIAamp DNA mini kit (QIAgen, Germantown, USA) according to the manufacturer's instructions.

**Figure 4:**
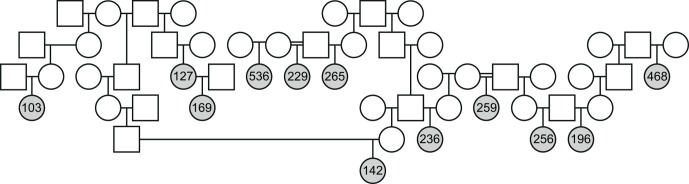
Pedigree showing the familial relationships of 12 of the 17 animals from the NEPRC colony. Legend: females are represented by circles and males by squares. Animals diagnosed with endometriosis are shaded in gray.

### 2.5 MHC typing

MHC typing of both class I and class II alleles was performed on DNA samples from these monkeys, namely for (*Macaca mulatta*) *Mamu-A1* (MHC class I, locus A1) and *Mamu-DRB* (MHC class II DR, beta-chain) by microsatellite (STR) typing with STRs D6S2854 and D6S2859, being *Mamu-A* specific, and D6S2878, being *Mamu-DRB* specific markers (Doxiadis et al., 2007, 2013). For animals from the NEPRC colony, additional high-resolution sequencing was done using Roche 454 technologies on blood-derived lymphocyte cDNA (Karl et al., 2013; Wiseman et al., 2013). In the case of the animals of BPRC, additional high-resolution Sanger sequencing had been performed beforehand and published previously (Otting et al., 2005; Doxiadis et al., 2013). Since the animals were members of breeding colonies, kinship coefficients and/or pedigrees of the animals are known, and some MHC haplotypes could be defined as well by segregation analysis. Significance was determined by comparing the number of carriers of the haplotype with endometriosis to the number of carriers in the colony (colony size: BPRC, n=1383; NEPRC, n=380, colony frequencies shown in Table 2b) using a Fisher's exact test with Bonferroni correction for multiple testing (each of nine independent MHC *A1* alleles).

## 3 Results

### 3.1 Demographics of endometriosis cases

Eight female rhesus macaques from BPRC (ranging from 12 to 21 years of age, mean 17.0 years) and seventeen female rhesus macaques from NEPRC (14 to 20 years of age, mean 15.9 years) were identified with endometriosis based on clinical and histologic diagnoses (Figs. 1, 2, and 3). The mean body weight of NEPRC macaques with endometriosis was 9.93 kg, and for BRPC macaques it was 7.78 kg. Four of the 17 macaques with endometriosis from NEPRC had caesarean sections, while none of the macaques at BPRC had undergone surgery (Table 1).

### 3.2 Pedigree analysis

The relationship status of the animals that had been identified with endometriosis was determined in an attempt to first identify obvious Mendelian segregation and to identify confounds in association analysis that may result from cryptic genetic substructure within endometriosis cases compared to the colony as a whole. Among the eight monkeys identified at BPRC, there were two pairs of siblings. Among the seventeen rhesus macaques with endometriosis from NEPRC, there was one mother–daughter pair, one pair of half-sib, and one trio of half-sibs. Additionally, there were four more distantly related animals identified with endometriosis (Fig. 4, gray shading). Given the breeding patterns within the colony and the animals for which tissue was available and for which pathology could be ascertained, the relationships among the affected females were not different from random samples using bootstrapping.

### 3.3 MHC typing

Association analysis for endometriosis was robust to allele sharing between the animals. The rhesus macaques from BPRC (n=8) and NEPRC (n=17) underwent MHC typing for their *Mamu-A1* and *Mamu-DRB* alleles (Table 2a). The BRPC endometriosis cohort included the following MHC I (*Mamu-A1*) alleles: A1*001 (33.3 %), *002 (13.3 %), *004 (6.7 %), *007 (6.7 %), *008 (26.7 %), and *011 (6.7 %) (Table 2b), while animals with endometriosis from NEPRC had the following *Mamu-A1* alleles: A1*001 (9.4 %), *002 (12.5 %), *003 (3.1 %), *004 (15.6 %), *007 (21.9 %), *008 (21.9 %), *012 (6.3 %), and *026 (9.4 %) (Table 2b). The allele frequencies in BPRC endometriosis samples compared to controls revealed significant enrichment of Mamu-A1*001 (33.3 vs. 11.6 % in healthy animals, p= 0.007) in monkeys with endometriosis (Table 2b). In the NEPRC cohort, the MHC allele Mamu-A1*007 was significantly overrepresented in diseased macaques compared to controls (21.9 vs. 6.7 %, p= 0.003). These associations are not shared between the colonies. The Mamu-A1*026 allele is marginally overrepresented in the NEPRC colony (9.4 vs 1.5 %), although this does not pass the multiple testing correction. This allele is uncommon in both colonies and is only seen in the affected mother–daughter pair at NEPRC. Additionally, the *Mamu-DRB* haplotype, which is characterized by the DRB*W3:03 allele, may be overrepresented in diseased animals of the NEPRC colony (Table 2a) (17.64 vs. 3.74 % in healthy animals of BPRC). Although *Mamu-DRB* typing is not routinely performed at NEPRC, the comparison to the colony frequencies at BPRC may be relevant, since allele frequencies of *Mamu-A1* in the two colonies are comparable (Table 2b). Nevertheless, while this is putatively suggestive and warrants further study, it cannot be interpreted with certainty.

## 4 Discussion

In this paper, we report significant higher frequency of two *Mamu-A1* MHC class I alleles in rhesus macaques with endometriosis from two different primate centers, Mamu-A1*001 in BPRC macaques and Mamu-A1*007 in NEPRC macaques. The familial relatedness of several macaques with endometriosis from the two colonies supports a hereditary risk for this disease in rhesus macaques which is similar to that seen in women (Bischoff and Simpson, 2004; Ishii et al., 2003; Kennedy, 1999). The different *Mamu-A1* alleles may reflect the different origins of the two colonies. Since diseased and control animals of both colonies are part of breeding groups, the *Mamu-A1* and *Mamu-DRB* alleles can be inferred from pedigree analysis to be identical by state but not by descent, and the higher frequencies observed in affected individuals are not attributable simply to kinship. Although NEPRC and most of the BPRC animals are of Indian origin, the founder animals of both colonies may be from different parts of India. Additionally, two of the macaques of BPRC are a mixed breed, Indian–Chinese or Indian–Burmese, and one animal is from Burmese origin. Although two different *Mamu-A1* alleles were overrepresented in macaques with endometriosis from the two facilities, the arguably more important interpretation may be that both colonies share a significant disease association with class I alleles. These results are comparable to humans, where higher frequencies of different MHC class I B alleles are described in endometriosis patients; a significantly higher frequency of HLA-B 54 and CW7 is observed in Japanese patients (Ishii et al., 2002), whereas a significantly positive association with endometriosis of HLA-B7 is defined by Kitawaki and colleagues (Kitawaki et al., 2002). In addition, some MHC II *Mamu-DRB* alleles were overrepresented in animals with endometriosis at BPRC; however, conclusions were limited by the low number of animals analyzed. These findings are consistent with the reported higher frequency of HLA-DRB1*1403 (Ishii et al., 2002) and HLA-DQB1*0301 in women with endometriosis (Ishii et al., 2003).

In our macaque study, *Mamu-B* alleles have not been analyzed. Since MHC alleles, in humans as in macaques (de Groot et al., 2014), are well known to be subjected to linkage disequilibrium, it is plausible that the observed disease associations are not caused by a specific *Mamu-A1* allele itself but may be due to certain alleles of adjacent loci such as *Mamu-B*. Likewise, the disease association with certain *Mamu-DRB* alleles may also be caused by linkage disequilibrium. Linkage disequilibrium with *Mamu-DRB* alleles would also explain why no disease association with *Mamu-DRB* alleles and endometriosis has been observed in other human populations (Roszkowski et al., 2005). Accordingly, Kitawaki and coworkers conclude that there is a certain HLA haplotype, namely HLA-A24-B*0702-Cw*0702-DRB*0101, which is linked to endometriosis susceptibility (Kitawaki et al., 2002). Further analysis of extended haplotypes in rhesus macaques will help to clarify these findings. It is important to interpret the present findings in rhesus macaques with caution, as the associated alleles may simply represent markers of associated haplotypes rather than causative variants themselves.

There may be several ways in which immune system surveillance, function, or dysfunction may contribute to or promote endometriosis (Ishii et al., 2002; Forte et al., 2014). As demonstrated in previous work, women with endometriosis exhibit altered or reduced innate and even adaptive immunity (Dmowski et al., 1981; Ota and Igarashi, 1993; Chiang and Hill, 1997; Khan et al., 2009). Additional studies suggest an autoimmune component to endometriosis (Eisenberg et al., 2012). Specific MHC I alleles may result in altered immune responses, leading to uncontrolled growth of stem cells, progenitor cells, and/or ectopic glandular tissue (Forte et al., 2014). Further investigation of spontaneous endometriosis in primates is warranted. The MHC typing results suggest the likelihood of a comparable genetic predisposition to endometriosis in women.

## 5 Conclusions

The MHC I allele overexpression in our macaque cohorts suggests a role for immune system on endometriosis pathogenesis. Further research is required to fully understand how these MHC I (*Mamu-A1*) alleles contribute to disease.

## Data Availability

All and sequences are publicly available at the IPD-MHC NHP database .
